# Anticholinergic effect of resveratrol with vitamin E on scopolamine-induced Alzheimer’s disease in rats: Mechanistic approach to prevent inflammation

**DOI:** 10.3389/fphar.2023.1115721

**Published:** 2023-02-02

**Authors:** Ahmed I. Foudah, Sushma Devi, Aftab Alam, Mohammad Ayman Salkini, Samir A. Ross

**Affiliations:** ^1^ Department of Pharmacognosy, College of Pharmacy, Prince Sattam Bin Abdulaziz University, Al-Kharj, Saudi Arabia; ^2^ Chitkara College of Pharmacy, Chitkara University, Rajpura, Punjab, India; ^3^ National Center for Natural Products Research, School of Pharmacy, The University of Mississippi, University, MS, United States; ^4^ Department of Biomolecular Sciences, School of Pharmacy, The University of Mississippi, University, MS, United States

**Keywords:** Alzheimer’s disease, dementia, herbal therapies, neurological disorder, resveratrol, vitamin E

## Abstract

The most common form of dementia, Alzheimer’s disease (AD), is characterized by gradual declines in cognitive abilities and behavior. It is caused by a combination of factors, including amyloid-β (Aβ) accumulation, acetylcholine (ACh) loss, oxidative stress, and inflammation. Phenolic compounds have a variety of health benefits, including antioxidant activities. Thus, the purpose of this study was to investigate how resveratrol (RES) alone and in combination with vitamin E affected rats with AD using scopolamine (SCO). Animals are categorized into groups; (i) control, (ii) SCO (1 mg/kg i.p.), (iii) SCO + donepezil, (iv) SCO + RES (50 mg/kg, p.o.), (v) SCO + RES (75 mg/kg, p.o.), (vi) SCO + RES (50 mg/kg + vitamin E 1 mg/kg, p.o.) for 17 days. In rats, studied behavioural (NOR and EPM) and biochemical characteristics. In addition, brain histopathology was examined to investigate any damage to the hippocampus and neuroprotection. SCO-induced changes in acetylcholinesterase, protein carbonyl, and TNF-α improved after resveratrol treatment. RES increased antioxidant levels, decreased SCO-induced lipid peroxidation, and reversed SCO-mediated changes compared with the drug donepezil. The results indicated that RES and vitamin E had nootropic action in the NOR and EPM tests, measured by the recognition index and the inflection ratio. This study supports the efficacy of RES as a preventive and treatment agent for AD. Vitamin E showed a synergistic effect on RES, which helps in managing cognitive impairment AD.

## Graphical Abstract

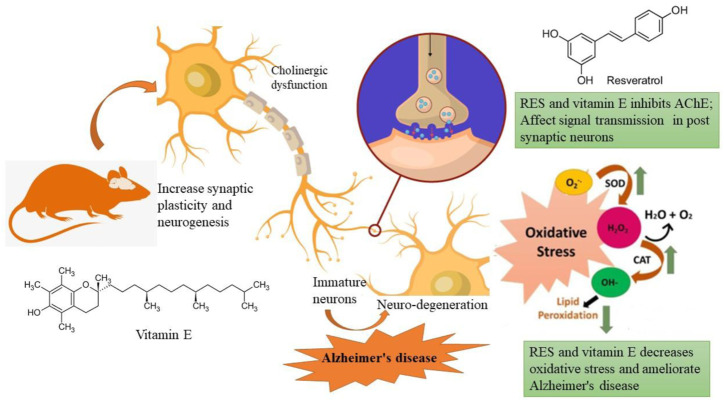

## 1 Introduction

Alzheimer’s disease (AD) is rapidly increasing and now affects more than 26 million people worldwide. There has been a twofold increase in the number of people living with neurological diseases (Birla et al., 2021). AD is the most common form of dementia, which causes long-term declines in memory and cognitive functions. In 2030, there will be 65.7 million dementia sufferers, and by 2050 there will be 115.4 million (Prince et al., 2013). It is known, however, that a number of risk factors have been associated with the development of this disease, as well as the gradual impairment of cognitive function. The development of this disease and the gradual impairment of cognitive functioning are known to be associated with a number of risk factors. Many factors, both genetic and environmental, are associated with the development of AD (Heneka et al., 2015; Onyango et al., 2021). It is still unknown how AD develops at the molecular level. As of now, two main theories have been proposed: the cholinergic theory and the amyloid cascade theory. The accumulation of amyloid-β (Aβ) peptides in the brain, hyperphosphorylated tau protein, and damage to neurons indicate the presence of AD (Randino et al., 2016; Long and Holtzman, 2019; Zhang et al., 2019). It is important to understand the role of oxidative stress in early stages of the disease in order to come up with another explanation of how the disease progresses. Free radical and oxidative stress theory suggests that neurodegeneration begins with oxidative damage. Therefore, protecting neurons from oxidative damage may delay or even prevent the onset of AD (Li et al., 2014). According to pathological theories, the decline of acetylcholine is responsible for memory loss and cognitive decline in AD (Cummings and Back, 1998; Ju and Tam, 2022).

**FIGURE 1:**
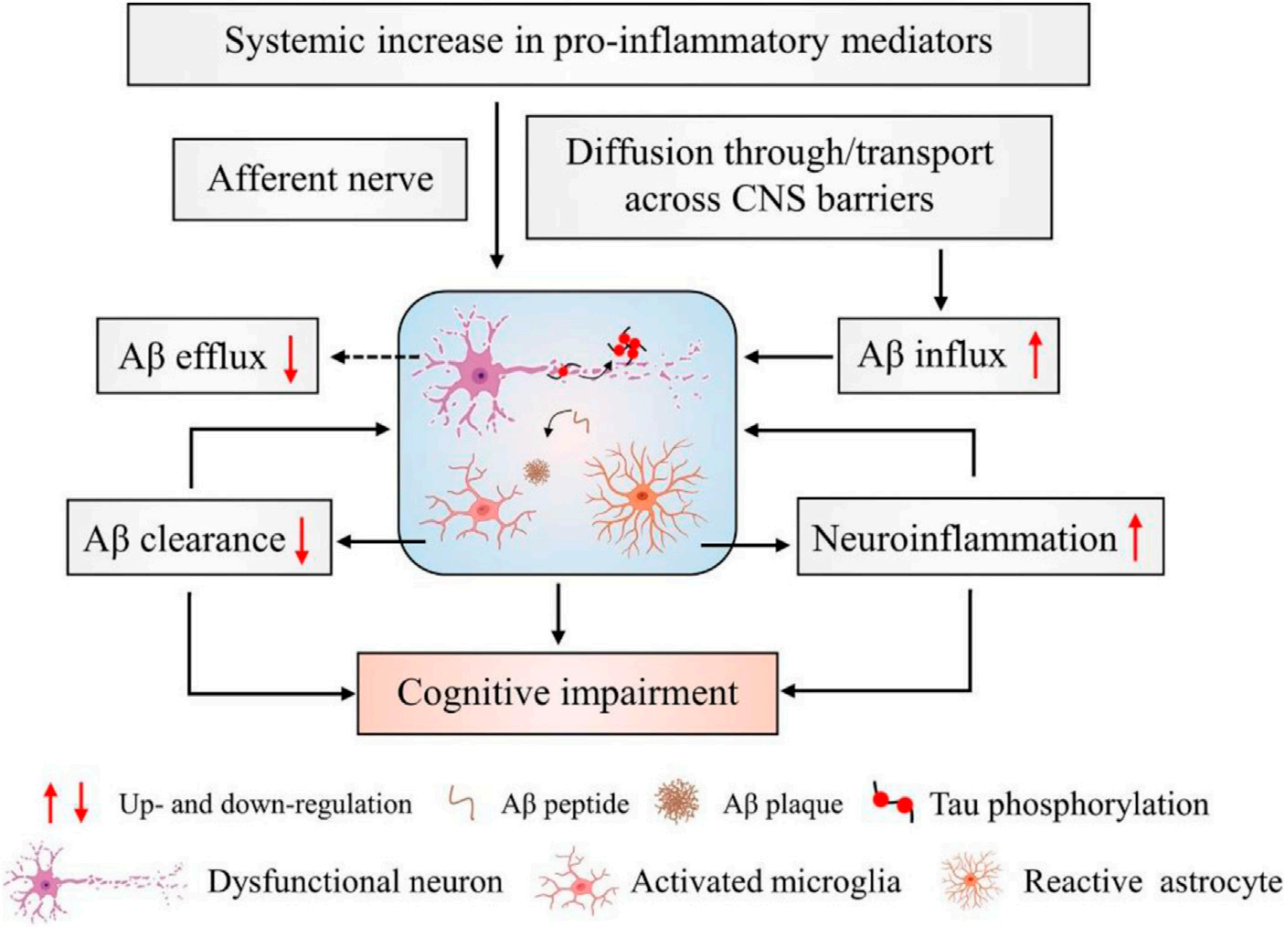
Inflammation and neurophysiology from AD. During the development and progression of AD, amyloid plaques activate microglia and astrocyte cells. These activated microglia increase the expression of inflammatory mediators such as interleukins, TNF-α, cytokines, etc., and enhance neuroinflammation. Thus, decreased Aβ clearance and increased neuroinflammation are responsible for cognitive impairment in AD.

Consequently, Alzheimer’s disease is based on protection or replacement. However, some medications may relieve symptoms for a short time. Acetylcholinesterase (AChE) inhibitors are currently the most effective agent for increasing cholinergic transmission and improving symptoms. Some approved AChE inhibitors, such as donepezil, galantamine, rivastigmine, etc., for treating AD. Treatment of AD and maintenance of acetylcholine levels (ACh) are best done with these drugs (Corbett et al., 2012; Bogdan et al., 2020). A number of studies have shown that inflammation in the brain leads to amyloid plaque formation and tau tangle formation, leading to the onset of AD and dementia. According to the data presented in Figure 1, increased levels of proinflammatory cytokines in the periphery do not lead to transient stimulation of neuronal death.

Since AD does not yet have a treatment that could reverse its effects, considerable research and development efforts to develop a therapy are urgently needed (Aisen et al., 2022). The drugs available today to treat AD only relieve symptoms and are associated with numerous adverse effects. Therefore, new pharmaceutical agents with neuroprotective properties are urgently needed to improve patient’s quality of life. Furthermore, discovering an effective therapy for Alzheimer’s remains a significant clinical problem. These molecules could be used alone or in combination with other treatments for AD (Ahmadi et al., 2021). Several medications can be used to prevent various symptoms of disease, and scientists around the world are working hard to find better treatments, ways to prevent disease, and, ultimately, a cure. Researchers are studying a variety of natural and synthetic phytoconstituents to determine if any of them might be beneficial in combating Alzheimer’s disease (Tohma et al., 2019).

In recent years, significant progress has been made in researching treatment therapies for Alzheimer’s disease using herbal medicines and active ingredients from natural herbal medicine. These agents can slow the progression of AD and improve the condition of the disease. The therapeutic potential of plant polyphenols ingested with food has been demonstrated by scientific research. The natural component known as polyphenol is regularly consumed and is attracting increasing interest due to its medicinal properties and other possible beneficial applications. Many plant species, such as grapes and berries, contain the secondary plant metabolite Resveratrol (RES; trans-stilbene), which plays the role of a phytoalexin (Zhang et al., 2020). Many studies have shown that RES has antioxidant properties, reduces inflammation, and slows cellular ageing, positively affecting cardiovascular disease, metabolic disease, inflammation, and cognitive function (Jang et al., 2021). Considering RES’s beneficial and pharmacological properties, we investigated the anticholinergic effect of RES as an anti-Alzheimer drug for treating and managing the disease *via* analysing behavioural and biochemical parameters. Studies have shown that food-derived vitamin E may be beneficial in preventing AD. There is some evidence that increased consumption of foods high in vitamin E could moderately reduce the risk of dementia and act as a protective agent against AD and cognitive decline. We therefore developed our hypothesis based on this literature, that vitamin E and another natural antioxidant may provide greater protection against Alzheimer’s than vitamin E alone. Therefore, we evaluated whether RES and vitamin E can improve AD symptoms synergistically.

## 2 Materials and methods

### 2.1 Chemicals

We purchased resveratrol, vitamin E, and scopolamine from Sigma Aldrich, United States, and donepezil from Alkem Laboratories Limited, Mumbai, India. Various chemicals were obtained from the regular chemical suppliers and manufacturers. Scopolamine (SCO) was dissolved in 0.9% sodium chloride (NaCl) solution and administered intraperitoneally (IPI). The chemical and solvent samples were all of the analytical grade and pure.

### 2.2 Selection of doses and grouping of animals

RES dosages (50 and 75 mg/kg) were selected based on oral acute toxicity experiments conducted by Williams et al. (2009). As per previous study, 1 mg/kg dose of vitamin E was selected (Shams et al., 2017). We divided the animals into groups of six, and administered the dose to each by oral gavage.

### 2.3 Experimental animals

**SCHEME 1:**
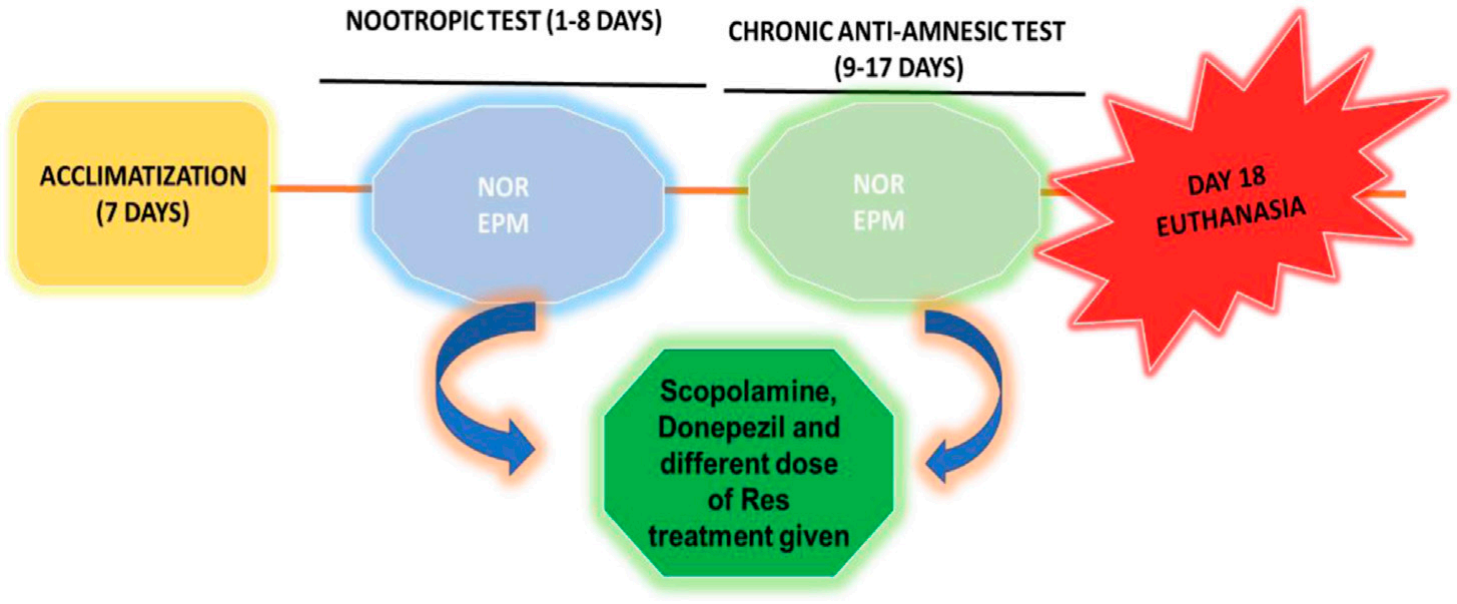
Workflow diagram of the experimental procedure.

In the present study, Sprague-Dawley rats weighing 160–190 g were used that were 8 weeks old, healthy, and male. The Bioethical Research Committee (SCBR-024-2022) of Prince Sattam Bin Abdulaziz College, Al-Kharj, Ministry of Education, Kingdom of Saudi Arabia, approved the studies. Each group consisted of six animals and had free access to food and water. The selected animals were housed and divided into different groups. In this investigation, dosages were provided in various ways depending on the delivered therapy, as shown in Table 1; Scheme 1. In all groups, SCO (1 mg/kg) was injected intraperitoneally for 9 days daily, causing amnesia following pretreatment with RES and vitamin E (day 9–day 17), except the control group. The Novel Object Recognition (NOR) task was performed on day 15 of the study, and the Elevated Plus Maze (EPM) task was performed on days 16 and 17 of the study, half an hour after the administration of scopolamine. The rats were sacrificed after the experiment was completed, and their brains were removed and examined histopathologically and biochemically.

**TABLE 1:** Grouping of animals.

Groups	Subjects	Treatment given
Group I	Negative control	Normal saline p.o
Group II	Positive control	SCO 1 mg/kg i.p. and normal saline p.o
Group III	Standard	SCO 1 mg/kg i.p. and donepezil 10 mg/kg p.o
Group IV	RES 50	SCO 1 mg/kg i.p. + RES 50 mg/kg p.o
Group V	RES 75	SCO 1 mg/kg i.p. + RES 75 mg/kg p.o
Group VI	RES 50 + vitamin E	SCO 1 mg/kg i.p. + RES 50 mg/kg p.o + vitamin E 1 mg/kg

### 2.4 Effect of resveratrol on the body weight change and organs weight in scopolamine-injected rats

During the 17-day therapy, rats were fed food that was measured and weighed, and each day the amount of food remaining in the cages was measured and gaged (rats were kept individually in cages). During the 17-day therapy, the body weight of the rats and various behavioural measurements were taken daily.

### 2.5 NOR in scopolamine-injected rats

The procedures and dimensions of the device NOR are the same as those developed by Bhuvanendran et al. (2018). The behavioural test was performed in a red-light environment between 9 a.m. and 6 p.m. Two clear culture bottles containing water and a Lego toy approximately the same size as one of the bottles (new object) were tested. During the test, two different types of objects were shown. Each of them has its size, colour and shape. Three phases are involved in evaluating these parameters: (i) habitualization, (ii) training of rats, and (iii) testing. On the first day of the experiment, rats explored an open field box without objects for approximately 10 min. Rats were placed in open fields for 5 min and allowed to examine two similar objects freely on the second day. A new item was introduced after 90 min of training, and the rats were tested for 2 min after that. The amount of time spent on each object was documented. For the open field boxes, ethanol (70%) was used between runs to eliminate scent trails. Using the following formula, we can calculate the recognition index:

Recognition index = TB/(TA + TB).

TA = time spent exploring the familiar object A; TB = time spent exploring the novel object B (Luine, 2015). An object is explored by smelling or touching it with the nose and front foot. Exploring objects by turning or sitting on them is not considered exploration (Bhuvanendran et al., 2018).

### 2.6 Elevated Plus Maze (EPM) in scopolamine-injected rats

To study many aspects of animal learning and memory, the EPM is a popular behavioural test. To conduct this experiment, minor adaptations were made to the methods used by Bhuvanendran et al. (2018). A medium-density fiberboard with a matte black acrylic surface is used to construct the EPM. This structure consists of two arms open (50 by 10 cm) spanning two arms closed with walls of 40 cm in height. The device was given a plus sign appearance by connecting these arms with a centre square (10 × 10 cm). The EPM was also raised 50 cm off the floor. This allows the labyrinth to be easily moved inside and outside the experimental room as needed and can be immediately moved to another location. We conducted the behavioural tests in the red evening light between 9 a.m. and 6 p.m. Memory was assessed using EPM in two separate sessions. The rats were positioned at the tip of an open EPM arm during the training phase. A stopwatch measured the so-called transfer latency (s), i.e., the time required for each rat to move entirely in one of the two closed arms. After each pass, 70% ethanol was used to disinfect the labyrinth to reduce the lingering scent. Each rat spent a total of 5 min in the labyrinth during each of the two training periods. Improvement in memory was thought to have occurred if the time required to transfer data during the test was reduced.

### 2.7 *In-vitro* assay for acetylcholinesterase activity

Without further modification, the method described above was used to perform the AChE activity (Berté et al., 2018). The rats in this experiment had their brains removed as soon as possible after decapitation and were placed in an inverted Petri dish. An isolated brain and hippocampus were homogeneously divided into ten volumes and weighed in a pH 7.2, 10 mM Tris-HCl buffer. After centrifuging the entire homogenate for 10 min, a slow-flowing supernatant was obtained. We centrifuged the hippocampal homogenate at 1,000 g for 15 min at 4°C in phosphate buffer (pH 7.0), and immediately used it for AChE analysis. Bovine serum albumin is the gold standard for determining the amount of protein present in various samples (Bradford, 1976).

### 2.8 Assay for lipid peroxidation

The reactive substance thiobarbituric acid (TBARS) in the hippocampus was tested using the technique described by Ohkawa et al. as a marker of lipid peroxidation (Ohkawa et al., 1979). In summary, a hippocampus homogenate in phosphate buffer (0.1 M, pH 7.4) with sodium dodecyl sulfate of 10%, w/v, for 10 min before adding 20% acetic acid. The reaction mixture had 0.8% thiobarbituric acid added to it, after which it was placed in a boiling bath and allowed to boil for 1 h. The quantities of TBARS were determined by the molar extinction values of 1.56 × 10^5^ M/cm, and the intensity of pink chromium was 532 nm.

### 2.9 Assay for protein carbonyl levels

As described by Bhatt et al. in their publication, protein carbonyls in the hippocampus were determined using a material called 2,4-dinitrophenylhydrazine (DNPH) (Bhatt et al., 2014). To homogenize the hippocampus, it was centrifuged at 11,000 × g for 15 min in 50 mM phosphate buffer with pH 7.4. The supernatants obtained from this procedure were employed in DNPH reactions. Using a 375-nm spectrophotometer, the absorbance difference between the samples treated with DNPH and those treated with HCl was discovered. We employed an aliphatic hydrazone with a molecular extinction coefficient (MEC) of 22.0 m/M/cm to estimate the amount of carbonyl content.

### 2.10 Estimation of TNF-α levels

All groups measured TNF-α levels in the blood. After fasting for 12 h, blood samples were taken from the retroorbital vein the following day. After the samples were placed in citrate tubes, they were cooled on ice and then centrifuged at a speed of 3,000 rpm for 10 min. This test is performed using quantitative sandwich enzyme immunoassay technology. A monoclonal antibody specialized in TNF-α was prefixed on a microplate. Wells are pipetted with standards and samples, and the immobilized antibody binds to any TNF-α present. A TNF-α-specific enzyme-linked polyclonal antibody was introduced into the wells after any unbound compounds had been washed away. The amount of TNF-α bound in the first phase was proportional to the colour development when a substrate solution was added to the wells and the wells were washed to remove unbound antibody-enzyme reagent. The colour development process was terminated, and the colour intensity was determined.

### 2.11 Histology of brain tissue

All rats were slaughtered under one-hour ketamine- and xylazine-induced anaesthesia after a behavioural experiment. To obtain a 5-µm-thick section, brain samples were first processed and then preserved in a paraffin block after being treated with 10% formalin. Hemolyte and eosin were used to stain the tissue sections. A bright-field microscope was used to examine the histological sections (Rajashri et al., 2020).

### 2.12 Statistical Analysis

A mean + standard error mean (SEM) is used to represent data. Graph Pad Prism soft-ware, version 7.04 (GraphPad Software, Boston, MA 02110), was used to test statistical significance, using one-way analysis (ANOVA) followed by a Bonferroni *post hoc* test. A *p* ≤ value of 0.05 was considered statistically significant.

## 3 Results

### 3.1 Effect of RES on the body weight change and organs weight in scopolamine-injected rat

This study investigated the impact of various doses of resveratrol *in-vivo* reports. As a result of injection or treatment of samples with SCO, body and internal organ weight are reduced, giving a simple and sensitive indication of toxicity. There was no statistical difference in the experimental group’s amount of food consumed and the change in body weight. The effects of SCO injection on body and organ weight were studied in mice (Table 2). We measured the initial body weights of the different groups before administering different doses of RES orally. During the trial, there were statistically significant variations in body weight between the different groups. Although there has not been a clear dose response, there have been significant differences between the three experimental doses. Therefore, 50 and 75 mg/kg showed the effect of reducing fat and weight. Surprisingly, the combination dose (50 mg/kg + vitamin E 1 mg/kg) significantly reduced animal body weight. Significant weight loss was observed when comparing the treatment groups with the control animals that were not treated. After sacrifice, the weight of the organs (liver, brain, and kidney) was not significantly different between all groups, indicating that the rats had no problems. As demonstrated in research, a link has been established between the dose of resveratrol and the reduction in animal body weight. The objectives of this research were (i) to investigate the effects of RES on scopolamine-induced Alzheimer’s disease in rats and (ii) to assess whether weight loss is associated with changes in the relative weight of the kidney, liver, and brain.

**TABLE 2:** Effect of RES on the body and organs weight in scopolamine-injected rats.

Measuring parameters (g)	Negative control	Positive control	RES 50	RES 75	RES 50 + vitamin E
Initial body weight (Day 1)	183 ± 4.33	174 ± 4.50	184 ± 3.21	184 ± 5.12	184 ± 4.08
Final body weight (Day 2)	179 ± 6.12	176.2 ± 5.15	182 ± 5.11	179 ± 5.45	168 ± 6.73
Brain	0.53 ± 0.05	0.53 ± 0.08	0.49 ± 0.03	0.51 ± 0.10	0.51 ± 0.07
Liver	5.42 ± 0.19	5.44 ± 0.16	5.24 ± 0.14	5.22 ± 0.16	5.26 ± 0.15
Kidney	1.16 ± 0.11	1.19 ± 0.09	1.12 ± 0.06	1.13 ± 0.10	1.11 ± 0.12

### 3.2 Effect of RES on nootropic activity in scopolamine-injected rats

**FIGURE 2:**
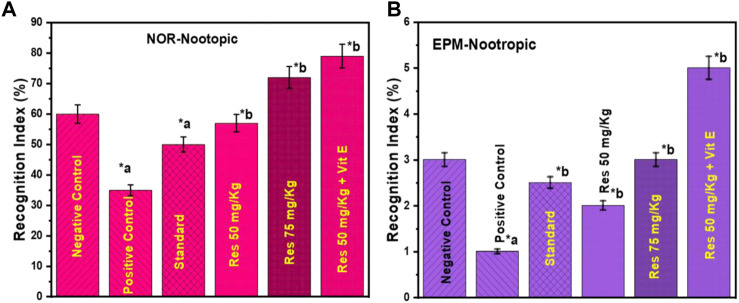
Behavioural analysis in rats **(A)** recognition indices in NOR for the nootropic model and; **(B)** inflection ratios in EPM for the nootropic model. ^a^ negative control vs. positive control; ^b^ positive control vs. treatment groups.

According to Figure 2 a, nootropic action was determined by conducting a NOR assay. The purpose of this study was to examine the effects of RES therapy on memory function after 7 days. The percentage of recognition index for the new object was calculated, and the results showed statistical significance with a value of *p* ≤ 0.05. In light of the results, groups pretreated with resveratrol showed a much more significant rise in the identification index for a new element compared to the control group and the donepezil-treated group.

The inflection ratio in the EPM was found to be considerably higher in the 50 mg/kg Resveratrol + vitamin E treated groups compared to the control group in the study, with a *p*-value of *p* ≤ 0.05 (Figure 2B). The 50 and 75 mg/kg doses did not show any statistically significant differences. Overall, this study suggests that RES has the potential to be a viable therapeutic agent for neuroprotection in the future. The nootropic benefits of RES are thought to be primarily due to its ability to decrease neuroinflammation and provide neuroprotection in the brain affected by neuroinflammation.

#### 3.2.1 Effect of RES on anti-amnesic activity in scopolamine-injected rats

**FIGURE 3:**
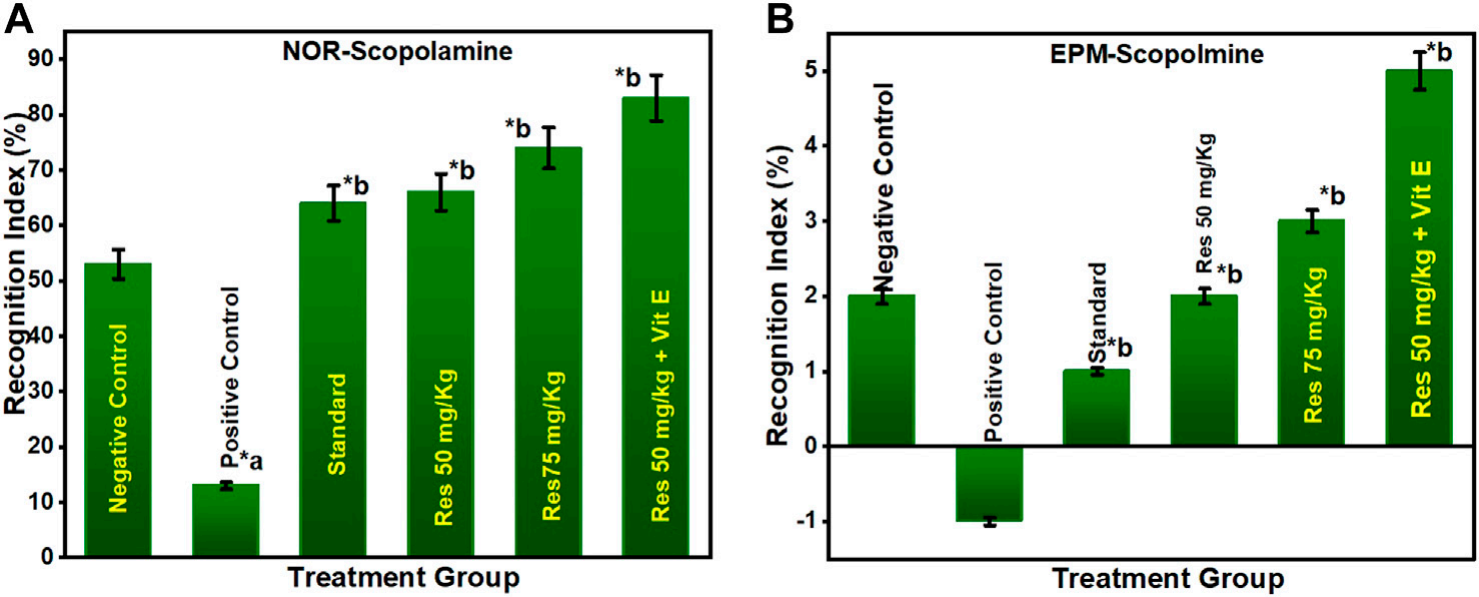
Behavioural analysis for NOR and EPM; **(A)** recognition indices in NOR for the scopolamine model; **(B)** inflection ratios in EPM for the scopolamine model. ^a^ negative control vs. positive control; ^b^ positive control vs. treatment groups.

As shown in Figure 3A, the percentage of recognition index decreased in the chronic SCO model. In addition, the percentage of recognition indices for the groups treated with 50 mg/kg of RES, 75 mg/kg of RES, and 50 mg/kg of RES with 1 mg/kg vitamin E was significantly higher than the group treated with donepezil (1 mg/kg). To study long-term spatial memory, researchers employ EPM, a behavioural test. The inflection ratio was used to determine whether there was an increase in memory retention in the embelin treatment groups compared to the negative control group in the EPM study (Figure 3B). The results of this investigation indicated that RES at doses of 50, 75, and 50 mg/kg RES + vitamin E had nootropic action in the NOR and EPM tests, respectively, measured by the recognition index and the inflection ratio. All the treated groups showed a significant difference in the percentage of recognition index compared to the negative group (*p* ≤ 0.05). This may be explained by the fact that higher doses increase the effectiveness of drugs, reaching their maximum effects. As a result, it can be inferred that the consumption of res polyphenols has a nootropic effect and can operate as a neuroprotective agent.

### 3.3 Effect of RES on acetylcholinesterase activity in scopolamine-injected rats

**FIGURE 4:**
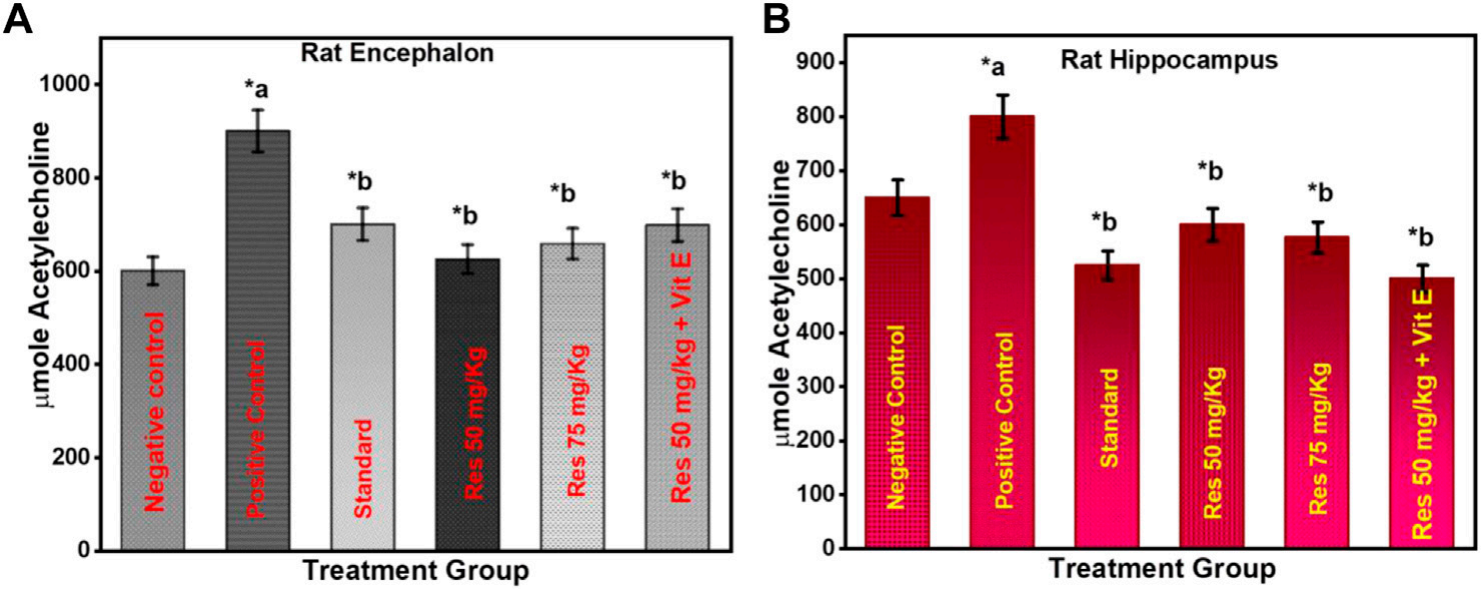
Effect of treatment on AChE activity in the animals **(A)** encephalon; **(B)** hippocampus. ^a^ negative control vs. positive control; ^b^ positive control vs. treatment groups.

The level of AChE is one of the most reliable, sensitive, and specific indicators of inflammation. In neurodegenerative disorders, measuring their activity may be helpful in predicting their development, prognosis, and responsiveness to therapy. As a response to RES, AChE activity was measured in the rats' encephalon (Figure 4A) and hippocampus (Figure 4B). Based on increasing doses administered to rats, the data indicate that res substantially suppresses AChE activity exclusively in the hippocampus. The results of this study reveal that RES can help improve cognitive function by decreasing the activity of acetylcholinesterase in the body. Acetylcholine is a neurotransmitter that helps the body process information. These findings indicate that RES can restore cholinergic system enzyme function. AChE activity is a biomarker of human cells, and lower levels are generally associated with older red blood cells in humans (Bhatt et al., 2014). In all RES-treated groups and the negative group (*p* ≤ 0.05), there was a significant difference in the percentage of recognition index.

### 3.4 Effect of RES on the lipid peroxidation levels in the hippocampus in scopolamine-injected rats

**FIGURE 5:**
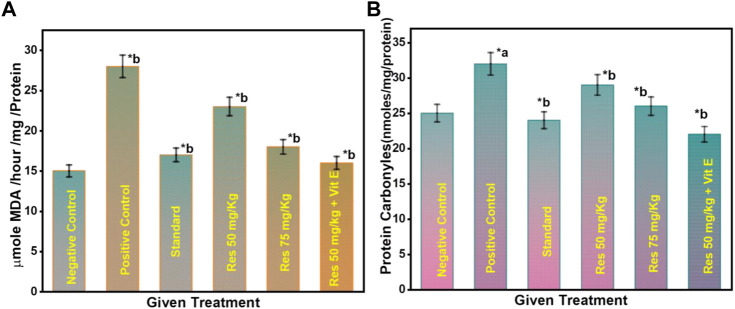
**(A)** Effect on the level of lipid peroxidation in the hippocampus of rats; **(B)** Effect on the protein carbonyl level in the hippocampus of rats. ^a^ negative control vs. positive control; ^b^ positive control vs. treatment groups.

Lipid peroxidation is believed to be a prevalent and particularly detrimental form of neuronal oxidative stress, causing membrane damage and the formation of many secondary products (Rajashri et al., 2020). On exposure to scopolamine injection in rats, lipid peroxidation increased significantly compared to the control (Figure 5A). In rats, 50 mg/kg RES + vitamin E significantly reduced high lipid peroxidation. As a result of these findings, it was revealed that superoxide inhibition has a dose-dependent direct connection with the concentration that causes the most excellent suppression of free radical formation. This can be occurred due to the anti-inflammatory effect of vitamin E. There was a significant difference between the RES treated and the negative group (*p* ≤ 0.05) in terms of recognition index. Data suggest that increased antioxidant defence and decreased lipid peroxidation are associated with RES’s ability to protect against the production of SCO-induced neuronal oxidative stress.

### 3.5 Effect of RES on the protein carbonyl levels in the hippocampus in scopolamine-injected rats

Furthermore, as seen in Figure 5B, the number of protein carbonyls in the hippocampus was reduced in the RES-treated group compared to controls. On the contrary, the administration of scopolamine resulted in a considerable increase in the quantity of protein carbonyl in the hippocampus compared to controls. The reduced free radicals resulted in a considerable reduction in oxidative damage to proteins in treated rats. A significant difference in the percentage of recognition index was found among the RES treated and the negative group (*p ≤* 0.05). These findings reveal that RES can have favourable effects on the redox state, suggesting that the intake of RES could improve the ability of neurons to cope with oxidative stress as they age.

### 3.6 Effect of RES on the TNF-α levels in the hippocampus in scopolamine-injected rats

**FIGURE 6:**
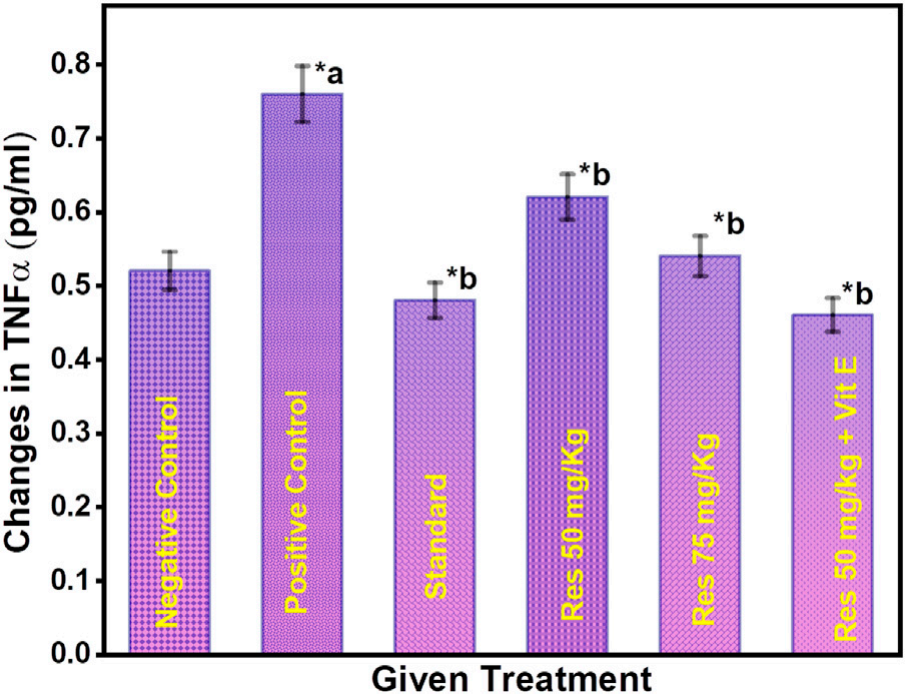
Changes in TNF-α levels after different treatments. ^a^ negative control vs. positive control; ^b^ positive control vs. treatment groups.

The concentration of TNF-α serum in sleep apnoea and narcoleptics was significantly increased compared to the normal control. Significant differences in serum TNF-α concentrations were observed between different groups (Figure 6). The AD group had a significantly higher TNF-α level than other treatment groups (*p* ≤ 0.05). TNF-α is a cytokine that plays an essential role in systemic inflammation and has been associated with disorders including Alzheimer’s and Parkinson’s (Prall et al., 1998). Furthermore, the positive control group have a high level of TNF-α in the brain. But, in RES and vitamin E treatment groups, TNF- α level was found to be reduced. Vitamin E can potentiate the anti-inflammatory effect of RES *via* attenuating TNF- α.

### 3.7 Effect of RES on the histology of the brain in scopolamine-injected rats

**FIGURE 7:**
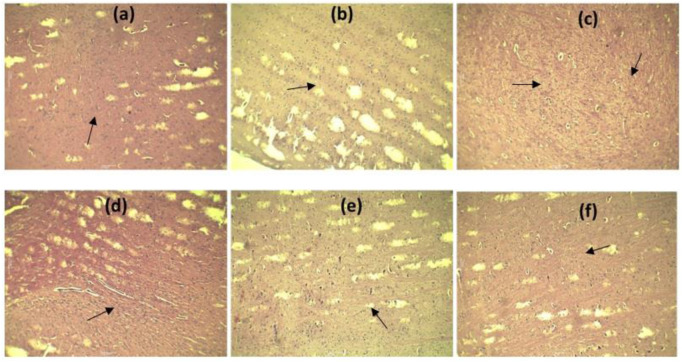
Effect of RES on the cortical regions of brain histology: **(A)** Negative control; **(B)** positive control; **(C)** standard; **(D)** RES 50 mg/kg; **(E)** RES 75 mg/kg; **(F)** RES 50 mg/kg + vitamin E.

To demonstrate the protective effects of neurons, brain tissue samples were collected, and tissue H&E stains were applied. There were no signs of neurodegeneration in control rats. Administration of rats with RES (50 and 75 mg/kg) and RES 50 mg/kg + vitamin E attenuated neuropathological changes in the brain (Figure 7). Relative therapy improved hippocampal damage and neuroprotection. The density of viable neurons has been significantly increased by RES dose (50 and 75 mg/kg) and RES 50 + vitamin E in the cortex that were subjected to scopolamine. A substantial increase in cortical neuron density was observed with the dose of RES 50 mg/kg + vitamin E compared to RES 50 mg/kg in treated rats*.*

## 4 Discussion

A neurodegenerative disease such as Alzheimer’s disease presents one of the greatest challenges to medical management because of the complexity of the pathophysiology. Herbal medicine and phytocomponents offer many methods to delay AD development and relieve symptoms. Low synaptic acetylcholine levels caused by the death of cholinergic neurons in the nucleus basalis magnocellular are thought to contribute to cognitive deterioration in Alzheimer’s disease. This has led to extensive research into raising ACh levels in synapses to create anti-dementia medications. One such tactic is the inhibition of acetylcholinesterase with donepezil, rivastigmine, and galantamine, which prevents the synaptic breakdown of ACh. Previous studies have shown that several phytoconstituents increase cholinergic activity in rats, including acetylcholine content and choline acetyltransferase activity. This may help explain some of the effects of these compounds on cognitive function and memory (Prall et al., 1998).

This study investigated the effects of RES on cognitive impairments in a rat model of dementia. RES alone and in combination with vitamin E were tested. As a result of the present study, we are able to shed light on the effects of RES on SCO-induced cognitive impairment in rats. In addition, it was shown that the combination of RES with vitamin E appears to have a synergistic effect. The results also suggest that these benefits are partly due to neurogenesis in the hippocampus *via* maintenance of the central cholinergic system.

As a first step, we investigated the effect of RES on abbreviated spatial and non-spatial memory functions in animals using the new object recognition test and the elevated-plus-maze test, respectively. This was done because functioning memories in animals are thought to be impaired early in their development (Schippling et al., 2000). Administration of RES significantly increased learning ability in the Elevated Plus Maze compared with scopolamine treatment alone and donepezil treatment on the learning day. Although the difference between the RES group and the scopolamine group was not statistically significant, it shows that the RES group learned more than the scopolamine group. The memory performance of those who received donepezil or RES was significantly better on the day of memorization than those who received scopolamine alone as therapy.

The time it took a subject to move from the light-free zone to the closed arm was used as a measure for testing purposes in experimental models. The studies found that scopolamine caused a significant reduction in functional memory performance in both tests; however, this effect was reversed by administering RES and donepezil to the participants. The reasoning behind this is that if a medicine has a favourable impact on learning, it will show up as a decrease in the latency to enter a dark room or a closed arm (Schippling et al., 2000). In animals, the response to resveratrol 50, 75, and RES 50 mg/kg + vitamin E were compared to positive and negative controls. According to the study, the TL/STL alterations in learning and retention trials show that resveratrol protein extract improves learning and memory.

RES acts on Ach receptors because it successfully counteracts the amnesic effects of scopolamine, a muscarinic receptor antagonist. The anti-amnesic effect of resveratrol may be due to its antioxidant property. A dose of 50 mg/kg of RES and vitamin E showed better efficacy than other doses, which are 25 and 50 mg/kg. Levels of Ach and TNF-α significantly restored by RES + vitamin E were near the donepezil (standard drug).

In contemporary times, natural compounds, especially those found in foods, have gained enormous attention in medicine due to their immense bioactivity. These bioactivities include antioxidant and anti-inflammatory properties (Cheng et al., 2010; Luo et al., 2022). The anti-inflammatory effects of RES and vitamin E have been demonstrated. The anti-inflammatory effect of RES was described by Zhou et al. (2018), who found that RES inhibited NF-κB signalling, thereby decreasing inflammation in animal models of acute pharyngitis. In addition, vitamin E has beneficial properties and can be used as an antioxidant in treating various diseases (Zhou et al., 2018). In recent studies, the anti-inflammatory effects of vitamin E have been studied in relation to atherosclerosis and other diseases (Muñoz and Munné-Bosch, 2019). According to research by Yang and Jiang (2019), vitamin E inhibits NF-κB, demonstrating its anti-inflammatory and anti-cancer effects in animal models. The anti-inflammatory effects of resveratrol and vitamin E work synergistically together (Yang and Jiang, 2019). Recently, the anti-inflammatory effects of vitamin E have been studied in atherosclerosis and other diseases (Muñoz and Munné-Bosch, 2019). Yang & Jiang (2019) indicated that vitamin E had anti-inflammatory activities by inhibiting NF-κB and anti-cancer effects in animal models (Yang and Jiang, 2019).

There is no doubt that the inflammatory response is crucial for the onset and progression of AD, but oxidative stress also plays a critical role. It is well known that Aβ-induced oxidative stress damages neurons and synapses by stimulating reactive oxygen species (ROS) and lipid peroxidation (Schippling et al., 2000; Cheng et al., 2010). In response, oxidative stress may increase Aβ synthesis. Because of these factors, it has been recommended that individuals with Alzheimer’s disease receive treatment with an antioxidant drug such as vitamin E (Gugliandolo et al., 2017). Vitamin E is vital for humans because it helps maintain healthy cell membranes. Vitamin E is a chain-breaking antioxidant in lipoproteins and cell membranes by inhibiting the oxidation of lipids and preserving the structural integrity of membranes. The development of many different organs and tissues, including the brain, depends on the presence of vitamin E, which plays a crucial role in various processes (Behl et al., 2022). Vitamin E has been shown to have multiple functions, including being a powerful antioxidant, a signalling molecule, and a gene-regulating molecule. It can also influence the action of membrane-associated proteins and incorporated proteins by modulating their signal transduction through molecular pathways independent of their antioxidant activity. This ability allows it to influence the action of membrane-associated proteins and incorporated proteins (Galli et al., 2017). In addition, the half-life of vitamin E in the brain is much longer than in other organs, which suggests the presence of specialized processes that regulate the central nervous system (Meganathan and Fu, 2016). Furthermore, the concentrations of vitamin E vary across various areas of the brain (Puri et al., 2022). Therefore, in the present study, the combination of vitamin E and RES was more beneficial than a high dosage of RES alone.

Histopathological images showed that RES and vitamin E treatment significantly improved the symptoms of Alzheimer’s disease. This study proved that RES, in combination with vitamin E has a good anti-Alzheimer’s effect in rats, which can benefit researchers, scientists and society. Moreover, vitamin E synergises with RES in Alzheimer’s disease due to its anti-inflammatory property.

## 5 Conclusion and future perspective

Scopolamine triggers Alzheimer’s-type dementia by increasing AChE levels, oxidative stress, and inflammation, which are closely associated with cognitive and memory problems. Studies show that the consumption of resveratrol reduces the risk of chronic diseases. Diverse herbal remedies have long been used around the world. RES and vitamin E are one of them. It has various pharmacological properties, including antioxidant, anti-inflammatory, antiproliferative, antibacterial, and neuroprotective effects. This study was carried out to determine the neuroprotective effects of resveratrol in rats exposed to scopolamine. RES reduced scopolamine-induced lipid peroxidation compared to donepezil and reversed scopolamine-mediated changes. Therefore, RES, a polyphenolic compound, and vitamin E may be a potential candidates for treating AD. Our results demonstrate that RES is a good candidate for treating AD pathology and vitamin E potentiates its effect.

There are many nutritional ingredients that are biologically active and can enhance the health and wellbeing of the body (such as RES, vitamin E, etc.,). The role of these agents in human healthcare and its endurance is critical, especially in the development of future therapeutics. In the future, these agents could become a novel therapeutic agent in Alzheimer’s disease or other related conditions with adequate clinical evidence.

## Data Availability

The raw data supporting the conclusion of this article will be made available by the authors, without undue reservation.
